# Validating the Utility of Supervised Clustering Algorithm for Precise [^11^C]DPA-713 PET Brain Image Quantification

**DOI:** 10.2967/jnumed.124.268519

**Published:** 2025-05

**Authors:** Youjin Lee, Thanh D. Nguyen, Yong Du, Jennifer M. Coughlin, Sara A. Zein, Nicolas A. Karakatsanis, Sadek Nehmeh, Martin G. Pomper, Susan A. Gauthier, Yeona Kang

**Affiliations:** 1 Department of Mathematics, Pusan National University, Busan, Republic of Korea;; 2 Laboratory of Neuroimaging, National Institute on Alcohol Abuse and Alcoholism, National Institutes of Health, Bethesda, Maryland;; 3 Department of Radiology, Weill Cornell Medicine, New York, New York;; 4 Russell H. Morgan Department of Radiology and Radiological Science, Johns Hopkins University School of Medicine, Baltimore, Maryland;; 5 Department of Psychiatry and Behavioral Sciences, Johns Hopkins University School of Medicine, Baltimore, Maryland;; 6 Department of Neurology, Weill Cornell Medical College, New York, New York; and; 7 Department of Mathematics, Howard University, Washington, DC

**Keywords:** PET, [^11^C]DPA-713, neuroinflammation, multiple sclerosis, supervised clustering algorithm

## Abstract

The reliance of quantitative PET imaging on the arterial input function makes brain PET challenging to perform in certain populations, limiting the sample size. To address this challenge, a supervised clustering algorithm (SVCA) has been introduced as an alternative. Our objective was to validate SVCA’s performance for brain PET with [^11^C]DPA-713 that targets a putative marker of brain injury and repair. **Methods:** This study included a composite dataset comprising 12 healthy volunteers (HVs), with 6 participants from Weill Cornell Medicine and 6 participants from Johns Hopkins University School of Medicine. The minimum number of subjects required to define kinetic classes was identified. Next, the distribution volume ratio (DVR) was examined by comparing pseudoreference time–activity curves derived from SVCA (SVCA-DVR) with the conventional arterial input function–based DVR (AIF-DVR). Test–retest analysis was conducted to evaluate repeatability, considering volumes of interest (VOIs) of various sizes. Lastly, the research investigated differences in DVR values between the HVs and patients with multiple sclerosis. **Results:** The number of subjects necessary for the kinetic classes, which are critical to SVCA, was reduced to 7 from the existing minimum requirement of 10. This allowed for a more substantial independent validation within a defined dataset. Correlative analysis between SVCA-DVR and AIF-DVR demonstrated a strong relationship, with correlation coefficients of 0.86 for white matter and 0.95 for the thalamus. Furthermore, we noted a marked decline in absolute test–retest variability for SVCA-DVR, with reductions from 1.31% to 1.18% in white matter and 3.51% to 2.32% in the thalamus, relative to AIF-DVR. This pattern of reduced variability persisted across VOIs of disparate sizes, with the absolute test–retest variability remaining below 5% for SVCA-DVR, even in small VOIs (both high and low binding at 0.065 cm^3^). Analysis revealed a pronounced disparity in SVCA-DVR values of the thalamus when comparing HVs and patients with multiple sclerosis. **Conclusion:** The findings substantiate the pseudoreference time–activity curves derived from SVCA as a dependable and practical substitute for the quantification of [^11^C]DPA-713 PET scans of the brain.

## Visual Abstract

**Figure:**
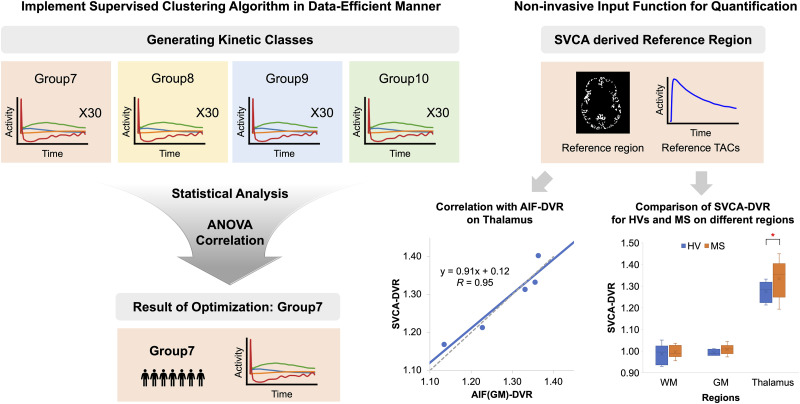

Neuroinflammation plays a pivotal role in the progression of neurodegenerative diseases, primarily through the activation of microglia and astrocytes (1*,*^2^). These cells, when activated, may adopt proinflammatory phenotypes that can contribute to the pathology of diseases such as Alzheimer, Parkinson, and multiple sclerosis (MS) (2*,*^3^).

The 18 kDa translocator protein (TSPO) is expressed on the outer mitochondrial membrane of glial (microglia and astrocytes) and vascular endothelial cells (4*,*^5^). Elevated levels of TSPO in distinct brain regions have been observed in select subtypes or stages of various neurologic conditions (6). Imaging TSPO with PET-based radiopharmaceuticals requires arterial blood sampling to determine the arterial input function (AIF), yet arterial catheterization is not always feasible (7*,*^8^). Consequently, there is growing interest in noninvasive approaches to estimate radiopharmaceutical binding to the target without arterial blood sampling. Several alternative approaches have been proposed, including the use of reference tissue methods, which eliminate the need for arterial blood sampling (9). However, these methods rely on the presence of an appropriate reference region devoid of specific binding sites, which may not be a feasible option for many neuroinflammatory or neurodegenerative diseases. In this context, advanced computational techniques such as the supervised clustering algorithm (SVCA) have been proposed for use in TSPO PET (10*,*^11^). These algorithms identify brain voxels with signs of negligible specific binding that can be used as a pseudoreference region.

[^11^C]DPA-713 has been applied in various central nervous system diseases (5). [^11^C]DPA-713 has been found to have advantages relative to other TSPO-targeting radiotracers that may position it to detect group differences in TSPO with more sensitivity (12–15). Although the application of the SVCA has been applied to PET data acquired using the first-generation ([^11^C]-(*R*)-PK11195 (10*,*^16^)) or select second-generation ([^11^C]PBR28 (17) and [^18^F]DPA-714 (18)) radiotracers for imaging TSPO, the SVCA has yet to be applied to data generated using PET with the second-generation TSPO radiotracer [^11^C]DPA-713.

This work aimed to assess the feasibility of the SVCA for quantifying regional TSPO levels in the human brain using [^11^C]DPA-713 PET data. Our objectives included measures of data efficiency, including a minimization of required sample size that was not considered in prior published studies of the SVCA method. The effectiveness of the SVCA was evaluated through comparative quantification analyses using the AIF and test–retest variability (TRV). The potential applicability of the SVCA for smaller regional and lesion-based analyses was also evaluated, through the examination of different volumes of interest (VOIs) and the addition of data from individuals with MS.

## MATERIALS AND METHODS

### Human Subjects

The study used a multi-institutional dataset, comprising 12 healthy volunteers (HVs) from Weill Cornell Medicine (WCM) and Johns Hopkins Medical Institutions (JHMI) (Supplemental Table 1; supplemental materials are available at http://jnm.snmjournals.org). At WCM, 6 HVs underwent brain MRI and 2 [^11^C]DPA-713 PET scans in a test–retest design with a 2-h interval. Meanwhile, 6 HVs from JHMI underwent a brain MRI and 1 [^11^C]DPA-713 PET scan. Additionally, 10 patients with MS from WCM underwent a brain MRI and 1 [^11^C]DPA-713 PET scan. The institutional review board of WCM and JHMI approved all procedures, and all subjects gave written informed consent. Patient characteristics and clinical data were obtained within 1 mo of the individual’s brain MRI and [^11^C]DPA-713 PET scan (Supplemental Table 1). Subjects were genotyped for TSPO (rs6971), only considering mixed-affinity binders and high-affinity binders in the study.

### [^11^C]DPA-713 Administration

For the PET studies, 526.4 ± 73.6 MBq (14.2 ± 1.9 mCi) of [^11^C]DPA-713 was administered through bolus intravenous injection, followed by flushing with 10–15 mL of saline solution.

### Image Acquisition and Processing

PET data acquisition began immediately after injection, lasting 90 min on the HRRT scanner (Siemens) at JHMI and the Biograph mCT (Siemens) at WCM. For HVs from WCM, the PET data were reconstructed into 32 frames (6 frames of 10 s each, then 4 × 30 s, 3 × 60 s, 2 × 120 s, 5 × 240 s, and 12 × 300 s) for a total scan time of 90 min. Meanwhile, for HVs from JHMI, the PET data were reconstructed into 30 frames (4 frames of 15 s each, then 4 × 30 s, 3 × 60 s, 2 × 120 s, 5 × 240 s, and 12 × 300 s) for a total scan time of 90 min. Data were reconstructed using the ordinary Poisson ordered-subsets expectation-maximization algorithm. JHMI used 6 iterations, 16 subsets, and 2-mm gaussian filtering, whereas WCM used 4 iterations, 21 subsets, and 4-mm gaussian filtering, with corrections for decay, dead time, attenuation, scatter, and randoms.

### Arterial Blood Sampling

Continuous arterial sampling was performed at 15-s intervals for the first 10 min using an automated fraction collector, followed by samples at 20, 30, 45, 60, and 90 min. Each of the blood samples was weighed, counted using a Wizard automatic γ-counter (PerkinElmer), and the activity concentration was calculated. Blood samples drawn at 5, 10, 20, 30, 45, 60, and 90 min after injection were used to estimate plasma metabolite fractions via centrifugation and high-performance liquid chromatography. The blood time–activity curves were then corrected for metabolites and decay, producing a metabolite and decay-corrected AIF.

### MRI and Preprocessing

Each subject underwent a T1-weighted MRI scan. Interframe head motion correction was achieved by rigidly coregistering the individual dynamic PET frames to the last 10-min image set using PMOD (version 3.8; PMOD Technologies Ltd. (19)). The resulting dynamic image set was then rigidly registered to the T1-MR image set.

### SVCA

The SVCA method is a noninvasive approach that identifies pseudoreference regions in the brain with minimal specific binding, ideal for studies lacking invasive procedures or preliminary binding information (10*,*^11^). It operates in 2 primary phases. The first step involves defining kinetic classes from HVs. In our study, 4 regions of kinetics classes were considered: low-binding gray matter (GM), white matter (WM), blood, and high-binding GM (e.g., thalamus). Using PMOD software, dynamic PET scans were normalized, and extracted time–activity curves for each class of masks were obtained from FreeSurfer (20). The normalization process involves subtracting the framewise average and then dividing by the SD within a brain mask at the individual level. For the blood class, image-derived input function was used by summing the initial frames of the dynamic PET imaging and manually identifying the carotid artery to obtain the time–activity curves on PMOD.

In the second step, pseudoreference curves were extracted. For subjects independent of kinetic class development, each voxel in the normalized PET scan was evaluated for similarity to the GM kinetic profile. Following prior work (11), the GM ratio was calculated as the weight of GM relative to all kinetic classes. Voxels with a GM ratio greater than 0.9 were selected, forming the pseudoreference region. Pseudoreference curves were then derived by averaging the time–activity curves of these voxels from the original dynamic PET images.

### Sensitivity Analysis of Kinetic Class Composite Number

**FIGURE 1.**
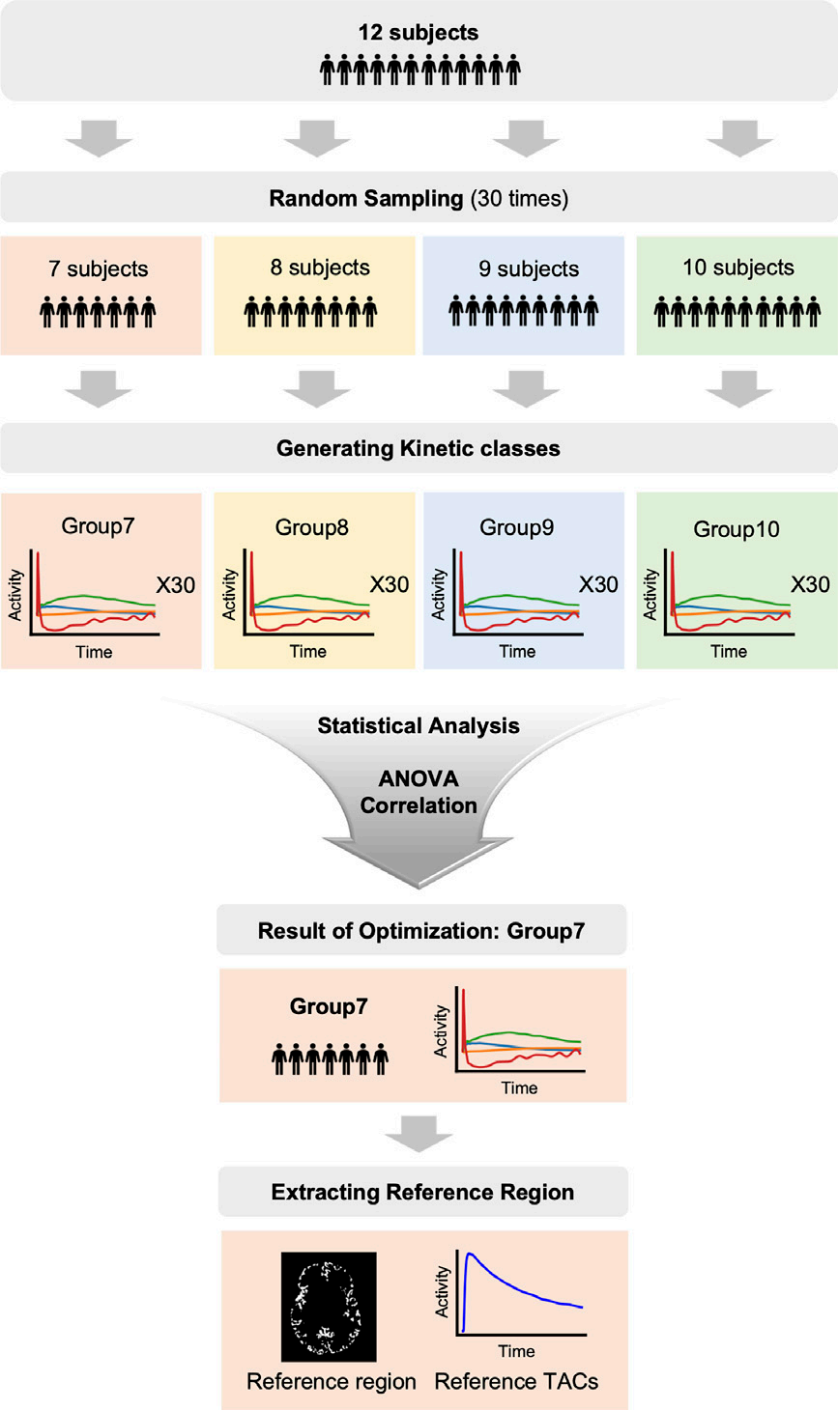
Comparison of pseudoreference curves generated from group 7 (orange) and group 10 (green). TAC = time–activity curve.

**FIGURE 2.**
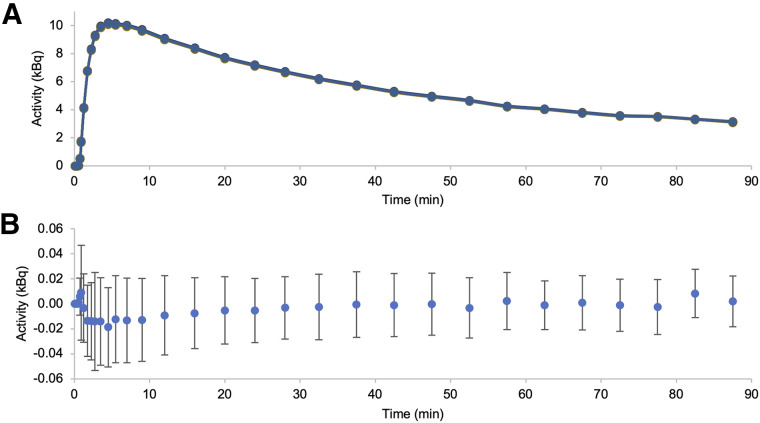
Comparison of pseudoreference curves for the left-out subject on leave-1-out validation. Full range of 10 pseudoreference time–activity curves using group 7 with mean of group 10 (A). Mean difference (group 7 − mean of group 10) is dotted with SD (B).

Schubert et al. (11) recommended at least 10 HVs to develop kinetic classes. To minimize the number of HVs for development, we compared kinetic classes derived from various subject counts, randomly selecting *N* of 12 (group *N*), as described in Figure 1. Group 10 served as the benchmark. ANOVA tests assessed differences between groups, and correlation coefficients were calculated against group 10. Using leave-1-out validation, we generated kinetic classes for groups 7 and 10 with 11 subjects, examining the robustness and accuracy of pseudoreference curves (Fig. 2).

### VOIs

Automated brain segmentation was performed on the MPRAGE MRI data using FreeSurfer version 8.0 (20). Standard FreeSurfer cerebral VOIs, including the thalamus, GM, and WM, were used for further analysis with PET images. For the patients with MS, normal-appearing WM masks were generated and replaced WM for patients with MS. To assess the influence of VOIs on parameter estimates, VOI masks of 0.065, 0.15, and 0.29 cm^3^ were generated, reflecting MS lesion sizes for evaluating the potential of the SVCA in lesion analysis.

### Measurement of Distribution Volume Ratio (DVR)

The reference Logan graphical model (LGM) (21) was used to calculate DVRs for each VOI with an equilibration time of 18 min using the SVCA-derived pseudoreference curve, which is denoted as SVCA-DVR. Meanwhile, the LGM (22) was used to calculate the total distribution volume for each VOI with a equilibration time of 18 min and a constant blood volume fraction of 0.05. Then AIF-DVR(GM) was defined as the ratio of LGM distribution volume of the VOI to that of the whole GM, and AIF-DVR(SVCA) was defined as the ratio of LGM distribution volume of the target to that of the pseudoreference curve. All kinetic analyses were performed using PMOD 3.5 (19).

### Statistical Analysis

To compare SVCA-DVR and AIF-DVR, the correlation coefficient, the intraclass correlation coefficient (23), and the Bland–Altman plot (24*,*^25^) were used. To assess the reproducibility of each method, the TRVs of SVCA-DVR and AIF-DVR were calculated for each subject, that is,

$$\mathrm{TRV}\;=\;100\times \left\{\frac{T\text{−}\mathrm{RT}}{\frac{\left(T\;+\;\mathrm{RT}\right)}{2}}\right\},$$ Eq. 1

where T and RT refer to values from the test and retest scans, respectively. Additionally, absolute TRV (aTRV) is determined by taking the absolute value of the TRV. The reliability coefficient (RC) values were calculated as using the following equation (26).

$$\%\mathrm{RC}\;=\;100\times 2.77\times \sqrt{\frac{1}{N}\sum_{j=1}^{P}\frac{\frac{{\left(T\text{−}\mathrm{RT}\right)}^{2}}{2}}{{\left(\left(T\text{+}\mathrm{RT}\right)/2\right)}^{2}}},$$ Eq. 2

The %RC score accounts for random and systematic errors, indicating real change with a greater than 95% probability (26). Statistical analyses were performed in MATLAB (R2021b; MathWorks) with significance set at a *P* value of less than 0.05.

## RESULTS

### Optimization of Subject Number Requirements for Kinetic Classes

An ANOVA test comparing kinetic classes (GM, WM, thalamus, blood) among groups 7–10 found no significant differences. Correlation analysis between group 7 and group 10 showed high coefficients, 0.992 (GM), 0.977 (WM), 0.982 (thalamus), and 0.976 (blood), indicating that kinetic classes constructed with 7 subjects are comparable to those with 10.

**FIGURE 3.**
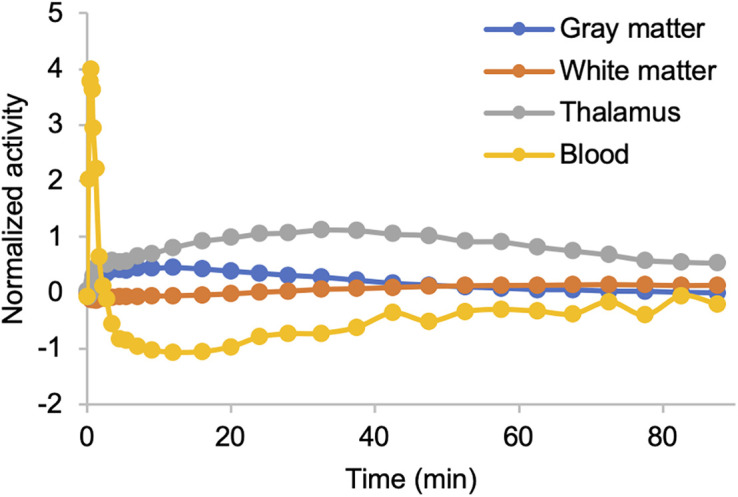
Developed kinetic classes based on 7 subjects.

We assessed the robustness and accuracy of pseudoreference curves. Curves from group 7 and group 10 showed similar trends (mean difference, −0.019 to 0.009 kBq; Fig. 2). This indicates that 7 subjects are sufficient to establish kinetic classes, formed by randomly selecting 3 subjects from WCM and 4 from JHMI, resulting in 4 classes: GM, WM, thalamus, and blood (Fig. 3).

### Comparison of SVCA-DVR with AIF-DVR

**FIGURE 4.**
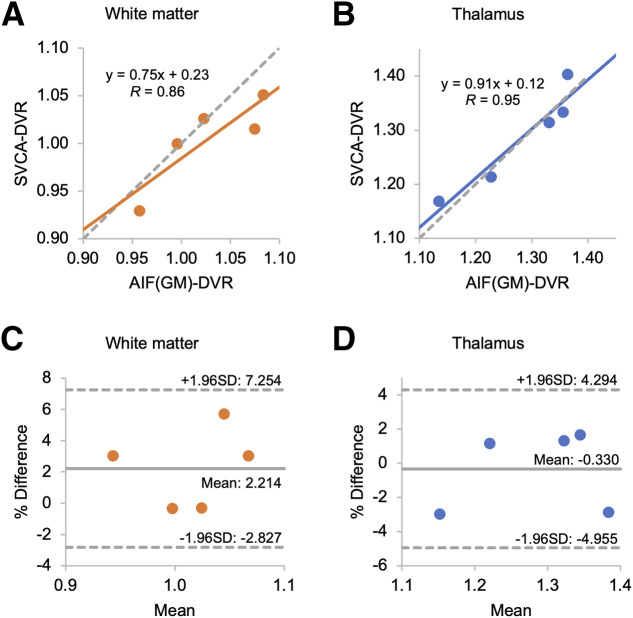
Correlation plot (A and B) and Bland–Altman plot (C and D) for DVR values from AIF- and SVCA-derived pseudoreference curves (*n* = 5).

Five subjects that were excluded in the development of kinetic classes were used for validation (2 from JHMI and 3 from WCM). We first compared AIF-DVR​(GM) and AIF-DVR​(SVCA). The correlation coefficients were 0.95 for WM and 0.98 for the thalamus, indicating strong agreement. Bland–Altman analysis further confirmed a high correlation between the 2 reference-based DVR values (Supplemental Fig. 1). In the subsequent analysis, we focused on AIF-DVR​(GM). Table 1 shows SVCA-DVR​ and AIF-DVR​(GM) values for WM and the thalamus. The correlation coefficients were 0.86 for WM and 0.95 for the thalamus, with consistent results across genotypes (mixed-affinity binders and high-affinity binders). Mixed-affinity binders demonstrated better agreement in both regions (Table 1). Intraclass correlation coefficient values between 2 DVR values were 0.82 for WM and 0.96 for the thalamus, indicating strong concordance between methods (Figs. 4A and 4B). Bland–Altman analysis (Figs. 4C and 4D) revealed a mean bias of 2.214% (WM) and −0.330% (thalamus), with all data within agreement limits, demonstrating robust method consistency for both regions.

**TABLE 1.** Comparison of AIF-DVR(GM) and SVCA-DVR (*n* = 5)

	WM				Thalamus			
Parameter	AIF-DVR(GM)	SVCA-DVR	*r*	ICC	AIF-DVR(GM)	SVCA-DVR	*r*	ICC
All	1.03 ± 0.05	1.00 ± 0.05	0.86	0.82	1.28 ± 0.10	1.29 ± 0.09	0.95	0.96
MAB	1.04 ± 0.06	1.03 ± 0.04	1.00	0.88	1.25 ± 0.16	1.25 ± 0.12	1.00	0.96
HAB	1.02 ± 0.06	0.99 ± 0.05	0.85	0.82	1.31 ± 0.07	1.31 ± 0.09	0.97	0.94

*r* = correlation coefficient; ICC = intraclass correlation coefficient; MAB = mixed-affinity binder; HAB = high-affinity binder.

### Test–Retest Variability of SVCA-DVR

To assess the SVCA repeatability in [^11^C]DPA-713 PET scans, we calculated SVCA-DVR and AIF-DVR(GM) values from test–retest scans of 3 validation subjects from WCM. The mean and SD of DVR values for WM, GM, and the thalamus are in Table 2 (DVR values for each subject are in Supplemental Table 2). Absolute variability decreased from 1.31% to 1.18% in WM and from 3.51% to 2.32% in the thalamus, indicating improved repeatability with SVCA. The %RC values for both regions were no greater than 7%, demonstrating robust repeatability. SVCA-DVR had even lower %RC, highlighting its reliability. Intraclass correlation coefficient values (0.98 for WM, 0.86 for the thalamus) confirm strong test–retest consistency.

**TABLE 2.** Descriptive Statistics and Absolute Variability, RC, and ICC for Test–Retest Study (*n* = 3)

	AIF-DVR(GM)					SVCA-DVR				
Parameter	Test	Retest	aTRV (%)	RC (%)	ICC	Test	Retest	aTRV (%)	RC (%)	ICC
WM	1.04 ± 0.07	1.02 ± 0.06	1.31 ± 0.94	2.97	0.98	1.00 ± 0.06	1.00 ± 0.06	1.18 ± 0.34	2.38	0.98
Thalamus	1.30 ± 0.07	1.31 ± 0.06	3.51 ± 0.80	7.00	0.65	1.29 ± 0.06	1.31 ± 0.05	2.32 ± 1.69	5.30	0.86

ICC = intraclass correlation coefficient.

### DVR Comparison with Various VOI Sizes

The impact of VOI sizes on DVR values were investigated. The average size of WM and the thalamus and the size of VOIs are provided in Table 3. For each VOI, SVCA-DVR data of both test and retest scans are calculated from DVR parametric map using the SVCA. Smaller VOIs increased the DVR values in high-activity regions (thalamus) and decreased them in low-activity regions (WM). However, the smallest WM VOI (WM3) showed higher noise sensitivity, partial-volume effects, and location-specific variability. aTRV and the TRV were computed to examine the reliability, suggesting that smaller VOIs tended to show greater variability, though the relationship was not strongly linear. In the test–retest study, the smallest VOIs (0.065 cm^3^) had aTRVs of 2.43% (thalamus) and 3.23% (WM). Intraclass correlation coefficient values exceeded 0.90 for all regions. The highest %RC values were 7.16% for the thalamus and 11.46% for WM, confirming repeatability for small VOIs in both high- and low-uptake regions, supporting their use in lesion analysis.

**TABLE 3.** Analysis for SVCA-DVR Values on Different Sizes of VOIs for Test–Retest Study (*n* = 3)

Region	Volume (cm^3^)	Test	Retest	aTRV (%)	TRV (%)	ICC	RC (%)
Thalamus	15.04	1.24 ± 0.07	1.26 ± 0.05	2.00 ± 1.19	−1.37 ± 2.14	0.90	4.35
Thalamus1	0.29	1.49 ± 0.19	1.51 ± 0.14	3.08 ± 2.19	−1.52 ± 3.71	0.96	7.16
Thalamus2	0.15	1.50 ± 0.19	1.51 ± 0.16	2.35 ± 1.34	−0.76 ± 2.82	0.98	5.16
Thalamus3	0.065	1.51 ± 0.22	1.52 ± 0.18	2.43 ± 1.35	−0.46 ± 3.07	0.98	5.29
WM	443.39	0.96 ± 0.05	0.97 ± 0.06	1.30 ± 0.60	−0.63 ± 1.51	0.97	2.71
WM1	0.29	0.86 ± 0.15	0.84 ± 0.10	4.08 ± 3.04	2.32 ± 4.83	0.94	9.61
WM2	0.15	0.85 ± 0.12	0.81 ± 0.10	4.73 ± 3.85	3.81 ± 4.96	0.92	11.46
WM3	0.065	0.99 ± 0.30	0.96 ± 0.31	3.23 ± 4.14	3.09 ± 4.27	0.99	9.62

ICC = intraclass correlation coefficient.

### DVR Comparison with HVs and Patients with MS

**FIGURE 5.**
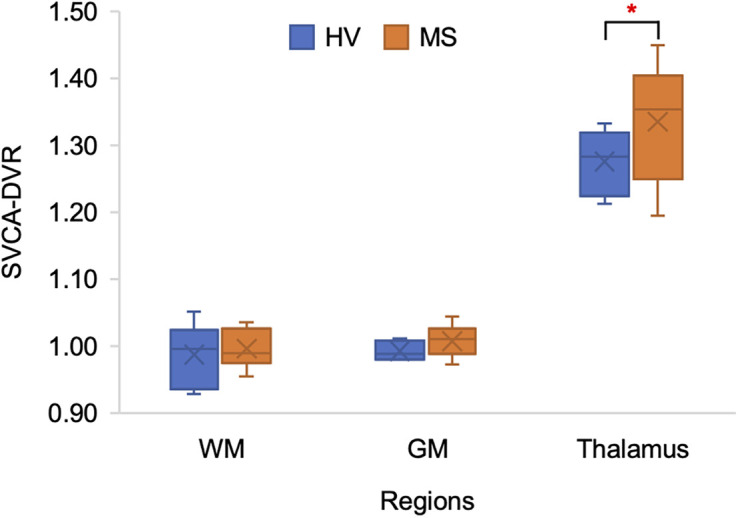
Box plots for DVR values from SVCA-derived pseudoreference curves (HV = 6, patients with MS = 10).

To evaluate the SVCA’s ability to differentiate HVs (*n* = 6) from patients with MS (*n* = 10), SVCA-DVR values were compared across GM, WM, and the thalamus. SVCA-derived pseudoreference curves showed no significant difference between patients with MS and HVs, except for 6 early time points before peak activity (Supplemental Fig. 2). Mean DVRs for HVs were 0.988 (WM), 0.993 (GM), and 1.276 (thalamus) and those for patients with MS were 0.996 (normal-appearing WM), 1.008 (GM), and 1.335 (thalamus). Significant differences were found only in the thalamus (*P* = 0.049), whereas WM and GM showed no significant differences (*P* = 0.693 and 0.126, respectively) (Fig. 5; Supplemental Table 3).

## DISCUSSION

TSPO expression in microglia and astrocytes increases in response to neurologic events such as trauma, infection, or conditions such as MS. PET imaging with radioligands ([^11^C]-(*R*)-PK11195, [^11^C]PBR28, [^18^F]DPA-714, and [^11^C]DPA-713) is commonly used to assess TSPO expression (27–29). However, its widespread brain distribution complicates the identification of a reference region, and blood sampling, often required for quantification, is not always feasible in clinical settings. To address this, the SVCA offers a noninvasive method for quantifying TSPO with PET imaging. This study is the first to apply the SVCA to human [^11^C]DPA-713 brain PET data from 2 research facilities.

The SVCA relies on predefined kinetic classes, typically developed using VOIs from at least 10 HVs (11). However, our findings demonstrate that kinetic classes from 7 subjects closely match those derived from 10, offering a viable alternative when fewer HVs are available (Fig. 2). This aligns with the [^18^F]DPA-714 study (18), which also used 7 subjects for kinetic classes, highlighting the SVCA’s effectiveness even with small cohorts.

To validate the SVCA, we assessed its efficiency by comparing test–retest variability against that of AIF-derived PET quantification, a recognized gold standard. Previous studies have used GM as a pseudoreference to quantify TSPO PET brain image including [^11^C]DPA-713 (17*,*^18^). In this study, we generated AIF-DVR​ values using 2 reference curves: GM and the SVCA pseudoreference. AIF-DVR(GM) and AIF-DVR(SVCA) showed strong agreement with a correlation coefficient greater than 0.9 for both WM and the thalamus (Supplemental Fig. 1). For consistency and benchmarking, we primarily used GM-based AIF-DVR to compare our SVCA-DVR values with established standards.

In past validation studies of the SVCA, the common practice involved using a leave-1-out strategy. For more rigorous validation, it is recommended that the validation set be entirely separate from the data used to develop the predefined kinetic classes. This ensures the robustness of the validation process. Previous methodologies (17*,*^18^) often incorporated mixed data usage, potentially leading to an overestimation of the method’s efficacy. In contrast, our study assessed the SVCA using an independent validation dataset, thereby enabling a rigorous evaluation of its generalization capabilities, distinctly separate from the dataset used for kinetic class creation.

Accurately assessing TSPO expression via PET requires determining radiotracer binding in brain VOIs, through either an AIF or a reference region. This study evaluated partial-volume effects and statistical influences on radiotracer binding in lesion-sized VOIs. VOI analysis revealed that smaller high-activity VOIs increased SVCA-DVR values with stable statistical uncertainty, whereas low-activity VOIs showed decreased SVCA-DVR values and higher uncertainty (Table 3). These findings align with [^11^C]-(*R*)-PK11195 studies (30), but [^11^C]DPA-713 demonstrated superior repeatability. For lesion-sized VOIs (∼7 cm^3^), [^11^C]-(*R*)-PK11195 had mean aTRVs of 18.62% (WM) and 3.21% (thalamus) (30), whereas [^11^C]DPA-713 achieved aTRVs of 4.73% (WM) and 2.35% (thalamus) on smaller VOIs (0.15 cm^3^). Test–retest analysis of SVCA-DVR showed improved repeatability and less variability compared with AIF-DVR(GM), with aTRVs below 5% even for VOIs under 1 cm^3^ (Table 3). These results highlight SVCA-DVR as a reliable, noninvasive method for longitudinal TSPO studies using the reference LGM.

From the comparative study of SVCA-DVR in patients with MS and HVs, we found that the thalamus of patients with MS showed a DVR significantly higher than that of HVs (Fig. 5; Supplemental Table 3). These results are consistent with numerous TSPO studies wherein the thalamus is the most consistent brain region to have elevated levels in patients with MS relative to controls (5). Moreover, the studies align with our results, showing no significance in GM. Although the studies reported a significant difference in WM between patients with MS and controls, we did not observe this difference, which likely relates to our small sample size. Additionally, there were significant differences of SVCA pseudoreference curves between patients with MS and HVs at early times. However, since the linearization in graphical analysis is applied at later time points during quantification, the difference at early time point has minimal effects on quantification.

This study validated the SVCA as a reliable and noninvasive method for quantifying [^11^C]DPA-713 PET imaging, though it has limitations. The assumption that kinetic classes from 10 subjects serve as a gold standard remains untested against larger datasets (e.g., 20–30 subjects). Data from 2 institutions, using different scanners and reconstruction methods, introduced potential bias. The limited data posed a constraint. Alternatively, we generated kinetic class libraries from mixed (primary), WCM (6 subjects), and JHMI (6 subjects) datasets. DVR​ values calculated using SVCA pseudoreference curves showed no significant differences across libraries with high nonsignificant levels (*P* > 0.85), suggesting minimal impact from scanner variability or lack of harmonization (Supplemental Table 4). These findings also align with those of García-Lorenzo et al. ([^18^F]DPA-714) (18). Although the SVCA has shown promise as a noninvasive method for quantifying TSPO, its limitations remain underexplored. Studies by Plavén-Sigray et al. (16) and Zanotti-Fregonara et al. (17) indicate that pseudoreference curves may still be contaminated by TSPO expression or overexpression, reducing sensitivity to subtle changes in tracer binding. This limitation could affect the technique’s accuracy in detecting small variations in TSPO levels. Blood–brain barrier disruption, observed in diseases such as MS, may contribute to increased tracer uptake including an early time point of reference curves in the patient cohort, warranting further investigation in future studies.

## CONCLUSION

This study confirms the SVCA’s effectiveness for [^11^C]DPA-713 PET quantification, offering data-efficient results. SVCA-generated pseudoreference curves present a reliable and noninvasive substitute for arterial blood sampling, enhancing the clinical applicability of TSPO imaging across a wide spectrum of neurodegenerative diseases. Furthermore, given its consistent variability across VOIs of different sizes, this method shows promise for broadening the clinical translation of TSPO to lesion-based studies of diseases such as MS.

## DISCLOSURE

Susan Gauthier has received research funding from NIH, NMSS, and Genentech and consulting fees from Biogen and Continuum Therapeutics. Youjin Lee has received funding from the National Research Foundation of Korea. No other potential conflict of interest relevant to this article was reported.

## Notes

KEY POINTS **QUESTION:** Can SVCA-derived pseudoreference curves serve as a reliable and noninvasive input function for [^11^C]DPA-713 PET? Can this be implemented efficiently? **PERTINENT FINDINGS:** SVCA-DVR shows a strong correlation with AIF-DVR in regional analysis and improved test–retest repeatability. It reliably distinguishes thalamic uptake between HVs and patients with MS, even in smaller VOIs. **IMPLICATIONS FOR PATIENT CARE:** Analysis of VOIs and discrepancies between HVs and patients with MS underscores the potential of small region or lesion analysis in clinical applications.
